# Supplementary material for: Exploring the Organic Acid Secretion Pathway and Potassium Solubilization Ability of Pantoea vagans ZHS-1 for Enhanced Rice Growth
Source: Plants (Basel). 2024 Jul 15;13(14):1945. doi: 10.3390/plants13141945 (PMC11281029; doi:10.3390/plants13141945)
Supplement: Supplementary file 1 [file plants-13-01945-s001.zip › plants-3032485-supplementary.pdf]

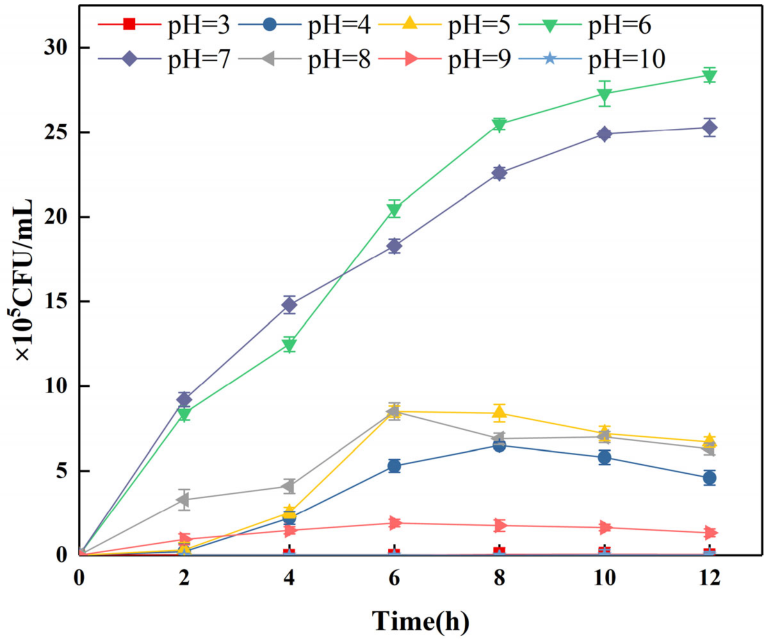

Figure S1. Growth curves of ZHS-1 in potassium solubilizing media with different pH values.

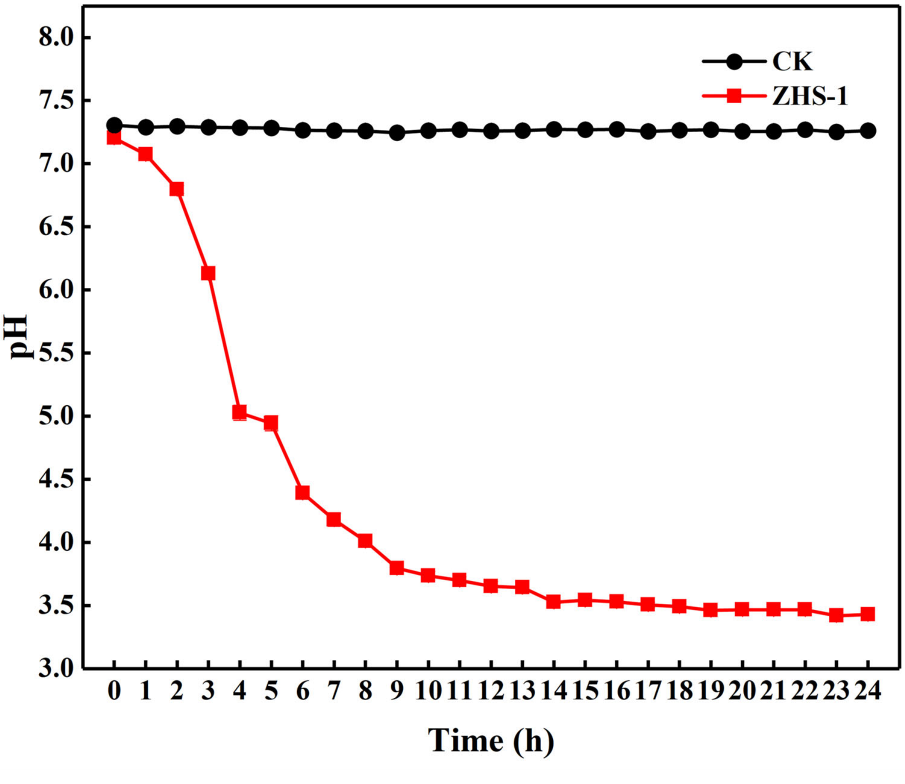

Figure S2. The pH changes of strain ZHS-1 in the potassium-solubilizing medium for 24 hours.

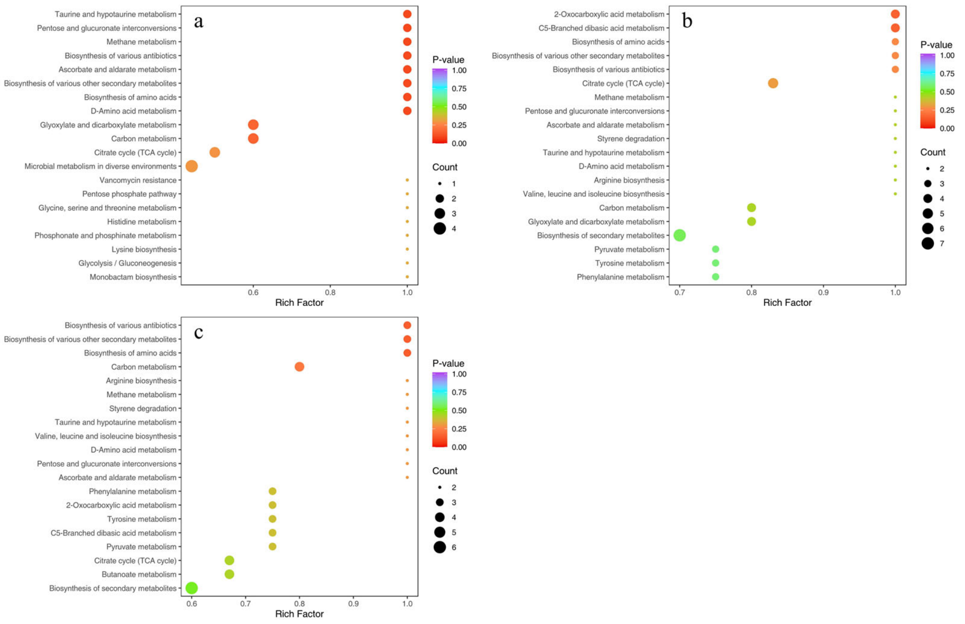

Figure S3. KEGG enrichment map of differential metabolites. (a) 2 hours vs. 4 hours KEGG differential enrichment bubble diagram. (b) 2 hours vs. 8 hours KEGG differential enrichment bubble diagram. (c) 4 hours vs. 8 hours KEGG differential enrichment bubble diagram.

Table S1: Phenotypic changes of rice seedlings under different treatments.

Seeing stage	Root length(mm)	Root surface area(mm2)	Root volume(mm3)	root tips number	root dry weight (g/plant)	Stem dry weight (g/plant)	Leaf dry weight (g/plant)
CK1	24482.4±432.67d	64755.65±48.46d	37219.7±26.11d	4940.5±53.03d	2.53±0.32c	4.27±0.74b	4.24±0.58b
CK2	31845.5±676.15b	119659±327.54b	101533.57±93.24b	6196±86.27b	5.25±0.27a	8.21±0.48a	6.43±0.57a
T1	27401.54±107.32c	78545.56±26.98c	54389.34±19.87c	5754.5±101.12c	4.43±0.22b	7.81±1.06a	6.08±0.45a

Table S2: Organic acid content of the sample.

Compounds	Concentration(ng/mL)		
	2h	4h	8h
pyruvic-acid	716.2966	423.8823	N/A
pantothenic-acid	506.043	416.0224	259.7008
oxoglutaric-acid	977.906	2213.81	16745.58
cis-aconitic-acid	3996.854	4619.62	8232.04
succinic-acid	4473.31	3351.66	4915.12
fumaric-acid	30.3065	16.66576	6.519822
3-D-hydroxybutyric-acid	N/A	N/A	42.50106
aminobenzoic-acid	N/A	N/A	0.362821
indole-3-acetic-acid	1.441868	4.382432	16.25348

L-malic-acid	205.9779	193.995	N/A
pyroglutamic-acid	1362.712	886.302	499.751
4-coumaric-acid	0.495418	0.751107	N/A
cryptochlorogenic-acid	N/A	N/A	14.22168
neochlorogenic-acid	N/A	N/A	1.860516
4-aminobutyric-acid	271.7522	282.2354	316.5156

Table S3: The chemical properties of the planting soil.

Treats	Total N TN (g·kg-1)	Available P AP (mg·kg-1)	Available K AK (mg·kg-1)	pH
Content	2.16±0.03	2.41±1.03	35.50±1.70	7.35±0.04
